# Study of the human hippocampal formation: a method for histological and magnetic resonance correlation in perinatal cases

**DOI:** 10.1007/s11682-023-00768-4

**Published:** 2023-04-06

**Authors:** Joaquín González Fuentes, Sandra Cebada-Sánchez, Maria del Mar Arroyo-Jiménez, Mónica Muñoz-López, Eloy Rivas-Infante, Guillermo Lozano, Francisco Mansilla, Francisca Cortes, Ricardo Insausti, Pilar Marcos

**Affiliations:** 1 Centro Regional de Investigaciones Biomédicas (CRIB), Avenida de Almansa 14, 02006 Albacete, Spain; 2 Department of Health Sciences, University of Castilla-La Mancha, School of Pharmacy, Albacete, Spain; 3 Human Neuroanatomy Laboratory, Department of Health Sciences, University of Castilla-La Mancha, School of Medicine, Albacete, Spain; 4 Servicio de Anatomía Patológica, Hospital Virgen del Rocío. Avenida Manuel Siurot, 41013 Sevilla, Spain; 5 Radiology Department, University Hospital, Hermanos Falcó, 02006 Albacete, Spain

**Keywords:** Human, Postnatal development, Hippocampal formation, MRI, Longitudinal axis

## Abstract

Little information is available on the magnetic resonance imaging (MRI) determination of the hippocampal formation (HF) during the perinatal period. However, this exploration is increasingly used, which requires defining visible HF landmarks on MRI images, validated through histological analysis. This study aims to provide a protocol to identify HF landmarks on MRI images, followed by histological validation through serial sections of the temporal lobe of the samples examined, to assess the longitudinal extent of the hippocampus during the perinatal period. We examined ex vivo MRI images from nine infant control brain samples. Histological validation of the hippocampal formation MRI images was obtained through serial sectioning and examination of Nissl-stained sections at 250 μm intervals along the entire length of the hippocampal formation. Up to six landmarks were identified both in MRI images and the serial histological sections. Proceeding in an anterior to posterior (rostrocaudal) direction, these were as follows: 1) the limen insulae (fronto-temporal junction); 2) the beginning of the amygdaloid complex; 3) the beginning of the lateral ventricle; 4) the caudal limit of the uncus, indicated by the start of the lateral geniculate nucleus (at the level of the gyrus intralimbicus); 5) the end of the lateral geniculate nucleus (beginning of the pulvinar); and 6) the beginning of the fornix. After histological validation of each of these landmarks, the full longitudinal length of the hippocampal formation and distances between landmarks were calculated. No statistically significant differences were found in total length or between landmarks. While the HF is anatomically organized at birth, its annotation is particularly challenging to perform. The histological validation of HF landmarks allows a better understanding of MRI images. The proposed protocol could be useful to assess MRI hippocampal quantification in children and possible variations due to different neurological diseases.

**Supplementary information:**

The online version contains supplementary material available at 10.1007/s11682-023-00768-4.

## Introduction

The hippocampal formation (HF) comprises the hippocampus (dentate gyrus and hippocampal fields CA1–3), subiculum, presubiculum, parasubiculum, and entorhinal cortex (EC). All these structures are located on the medial aspect of the temporal lobe, both in infant (Insausti et al., 2010) and adult brains (Insausti & Amaral, 2012; Insausti et al., 2010). These structures are linked by step-wise, largely unidirectional connections that likely play a central role in declarative memory of facts and events (Carr et al. 2010; Squire et al. 2004). Topographically, the HF starts at the beginning of the EC in the rostral part of the temporal lobe, usually a few millimeters behind the *limen insulae* (frontotemporal junction). The HF in the adult human brain extends caudally for about 5 cm, as far as the anterior limit of the calcarine sulcus (Insausti & Amaral, 2012). Here, the hippocampus turns dorsally and medially to form the fimbria–fornix transition, known as the *crus fornicis* or posterior pillars of the fornix (Franko et al., 2014). At a histological level, different HF fields appear progressively along a longitudinal axis. However, those fields are not easily recognizable in MRI along the rostrocaudal length of the HF.

The HF shows a rapid enlargement in volume from the perinatal period (birth and subsequent weeks), probably due to the increase in myelination during this period (Jabes et al. 2011). Likewise, human total brain volume experiences a rapid and nonlinear increase in childhood, reaching a maximum around puberty and adolescence (Knickmeyer et al., 2010; Suzuki et al., 2005).

The HF growth not only maintains a direct relationship with brain growth, but also with full-scale intelligence quotient, as shown in male children between 8–18 years of age. However, whether a larger hippocampal volume precedes formal education or is a result of it remains unknown (Schumann et al., 2007). Tests performed in children between 8–13 years of age show that a volume increase of the right CA3/dentate gyrus correlates positively with item–color accuracy (Lee et al., 2014).

The integrity of the HF structure and connectivity are crucial for normal cognitive development in children (Vargha-Khadem et al., 1997). Likewise, early HF and adjacent parahippocampal region lesions produce sustained memory deficit (Guderian et al., 2015; Vargha-Khadem et al., 1997). Cerebral hypoxia–ischemia may induce massive neuronal damage (Gonzalez Fuentes et al., 2021), leading to temporal lobe seizures, hippocampal sclerosis (Bernasconi et al., 2003), or memory deficits (Guderian et al., 2015; Vargha-Khadem et al., 1997; Zola-Morgan et al. 1986). Although most of these ischemic episodes take place during the perinatal period, when the hippocampal fields are histologically formed (Insausti et al., 2010), connections are still developing after this period, as seen in nonhuman primates (Ben-Ari, 2006; Jabes et al., 2011). A number of diseases, neurological and otherwise, can also disrupt the normal development of the HF. For instance, structural changes in the HF have been described in schizophrenia (Suzuki et al., 2005) and dyscalculia (Supekar et al., 2013).

Therefore, impairment of normal development in childhood may affect the HF volume, which, can generally be detected by MRI. Consequently, our study focuses on post-mortem, ex vivo MRI scans and their comparison with fine-grain cytoarchitectonic analysis of the HF fields of control brains at the perinatal stage. Our results indicate that several HF landmarks can be identified in MRI images, which can be validated by histological analysis. This allows a more precise measurement of the entire length of the HF, as well as HF segments defined by distances between landmarks. This approach might be used to assess MRI visible details to estimate HF length in vivo, therefore contribute to more reliable annotations of the hippocampus at the perinatal stage. This protocol would allow a clear identification of external temporal lobe landmarks throughout postnatal development in T1-weighted sequences performed with a 1.5 T magnet. Those values, gathered from a population of control cases, could be used as a reference for comparison with pathologic cases—i.e., memory and cognitive problems in children with perinatal HF damage.

## Methods

### Subjects

The study was performed using nine perinatal brain tissue samples (Table 1) from the Andalusian Public Health System Biobank (ISCIII-Red de Biobancos RD09/0076/00085), which were obtained from routine autopsies performed at the Pathology Department of the Virgen del Rocío Hospital (Seville, Spain) between 2003 and 2006. All the samples were free of neuropathological changes, and the cause of death was unrelated to neurological disorders and thus did not affect the brain. Data were anonymized by staff unrelated to the research team. According to Spanish law, consent was not necessary, although since 2004 written informed consent from the next of kin has been mandatory for any hospital or biobank using human samples for research. This research project—including detailed descriptions of objectives, brain manipulation methods, benefits and risks, data protection, and other issues—was conducted in accordance with the Declaration of Helsinki, Spanish legislation on research involving human samples, and the Spanish Data Protection Act—Organic Law 15/1999 and the updated Decree-Law 5/2018 on urgent measures for the adaptation of Spanish law to the regulations of the European Union on data protection, i.e., Regulation (EU) 2016/679 on the protection of natural persons with regard to the processing of personal data and on the free movement of personal data.

The study was approved by the local Clinical Research Ethics Committee of the University Hospital of Albacete, Spain (CEIC, session record number 10/06).

### MRI acquisition and histological brain tissue processing

Brains were removed and fixed following a protocol published previously (Insausti et al., 2010). To avoid artifacts and remove fixative excess, the brains were rinsed in distilled water prior to MRI scanning. T1-weighted images were acquired in a single 2D plane at 1 × 1x1 mm from each section using a Philips 1.5 Tesla Gyroscan Intera MR system at the University Hospital in Albacete (Spain) between 2005 and 2009, with the following parameters: spin echo-high resolution (SE-HR) sequences; echo time (TE) 15 ms; repetition time (TR) 450 ms; matrix 512 × 512; field of view 200 mm × 200 mm; voxel size 1 × 1 × 1; variable section thicknesses of 1.2, 1.5, or 2 mm; resolution: 1 mm. There is no slice gap since only 1 plane was acquired per section. Whenever possible, images were obtained in the coronal plane—orthogonal to the anterior–posterior commissure line (Insausti et al., 2010). An approximation was performed in cases where this line could not be explicitly determined. Different recording coils were used depending on the sample size, which in no case affected the scan resolution or the identification of the proposed landmarks. Some parameters such as acquisition time, angle, coils, or section thickness were adjusted to obtain the best resolution and image quality.

After performing MRI scans, the hemispheres that had been received intact (three cases) were cut into 1-cm-thick slabs, trying to keep to the same coronal plane as the MRI (Yushkevich et al., 2021). The temporal lobes were then dissected and placed into increasingly graded sucrose solutions (15% to 30%, in phosphate buffer 0.1 M, pH 7.4 at 4℃) for cryoprotection. Next, 50-µm-thick coronal sections were obtained using a sliding microtome coupled to a freezing unit (Microm, Heidelberg, Germany). The temporal lobes were serially sectioned so that the whole HF could be examined every 250 µm. The series was immediately mounted, Nissl-stained with 0.25% thionin, and analyzed histologically for identification of fields along the longitudinal axis of the HF. The sum of the total number of sections (including those damaged or discarded during the cutting process), multiplied by their thickness, yielded the anterior–posterior distance among histological landmarks. This information was translated into the MRI images, looking for correspondence and agreement between both series.

### Analysis and data collection

The analysis of the MRI series of images allowed the identification of six HF structures, which were later corroborated in Nissl-stained serial histological sections. These landmarks were as follows:

1. The limen insulae (LI) at the junction of the frontal and temporal lobes.
2. The beginning of the amygdaloid complex (A, start).
3. The lateral ventricle (LV) at the first section in which the temporal horn of the LV becomes visible, shortly after showing the rostral part of the subiculum.
4. The start of the lateral geniculate nucleus (LGN) at the most anterior part, which corresponds to the caudal end of the uncus, at the level of the gyrus intralimbicus*.*
5. The end of the LGN of the thalamus, at the level where the pulvinar (Pul) starts to get a rounded profile, separated from the more anterior portions of the thalamus.
6. The posterior pillars of the fornix (rus fornicis) on the fornix (Fx), adjacent and immediately rostral to the beginning of the splenium of the corpus callosum.

**Fig. 1:**
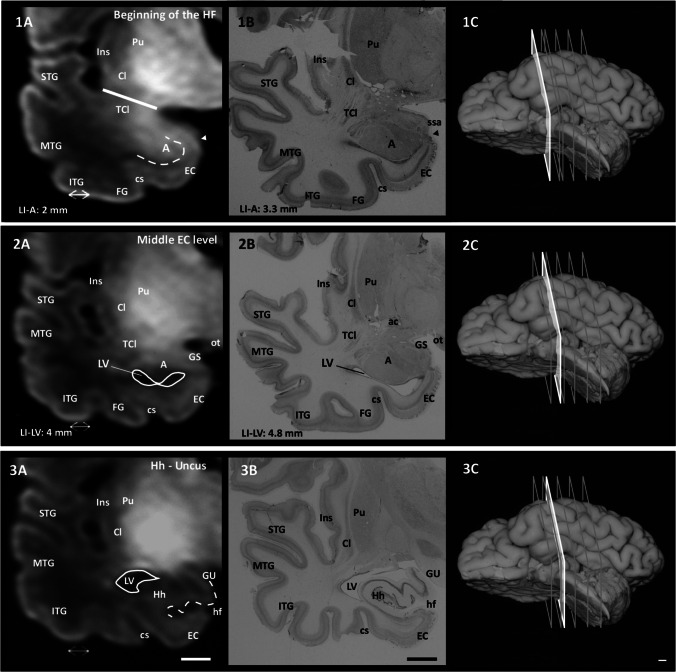
Rostrocaudal sequence of the levels and landmarks analyzed in this study taken from case 9. 1A: MRI image of the LI level. The straight white line shows the fronto-temporal junction, a few millimeters after LI and the beginning of the EC; this point is about 1 mm in front of the start of A (dashed line). 2A: MRI image of A. The solid line shows the outline of the LV. 3A: MRI image of Hh. The dashed line shows hippocampal digitations, while the solid line shows the outline of the LV. Images 1B–3B: histological sections corresponding to the same levels as in the left-hand side column (scale bar: 1 cm). 1C–3C: approximate levels (white section plane) in a ventrolateral image of one complete hemisphere (scale bar: 0.5 cm). Abbreviations: ac, anterior commissure; Cl, claustrum; cs, collateral sulcus; FG, fusiform gyrus; GS, gyrus semilunaris; GU, gyrus uncinatus; hf, hippocampal fissure; Hh, hippocampal head; Ins, insula; ITG, inferior temporal gyrus; MTG, medial temporal gyrus; ot, optic tract; Pu, putamen; STG, superior temporal gyrus; TCl, temporal claustrum

**Fig. 2:**
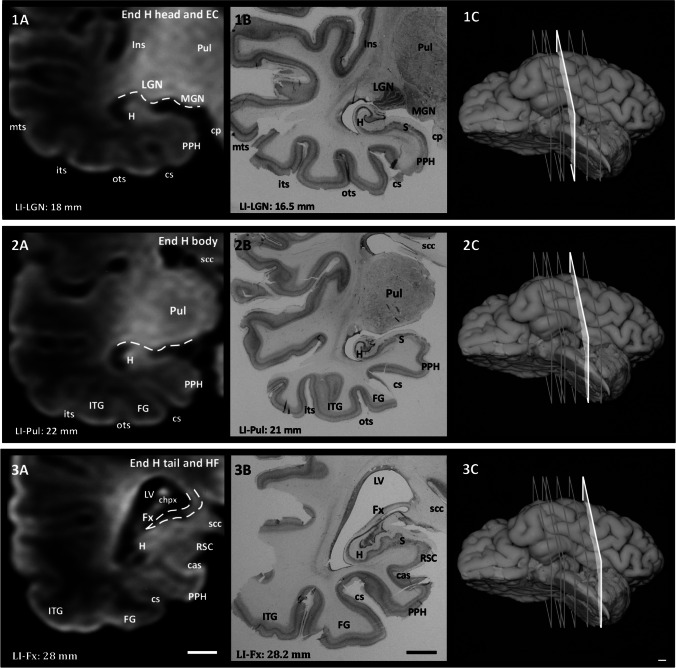
Rostrocaudal sequence of the levels and landmarks analyzed in this study taken from case 9. 1A: MRI image of LGN. The dashed line shows the surface of LGN and MGN in the LV. 2A: MRI image of Pul. The dashed line shows the separation between Pul and H. 3A: MRI image of Fx (dashed line). Images 1B–3B: histological sections corresponding to the same levels (scale bar: 1 cm). 1C–3C: approximate levels (white section plane) analyzed in a ventrolateral image of the whole hemisphere (scale bar: 0.5 cm). Abbreviations: cas, calcarine sulcus; chpx, choroid plexus of the lateral ventricle; cp, cerebral peduncle; H, hippocampus; its, inferior temporal sulcus; MGN, medial geniculate nucleus; mts, middle temporal sulcus; ots, occipito-temporal sulcus; PPH, posterior parahippocampal cortex; RSP, retrosplenial cortex; scc, splenium of the corpus callosum

The different landmarks can be appreciated in Figs. 1 and 2.

These landmarks were chosen because they were visible in all cases on MRI images and could be validated in Nissl-stained sections. Other brain regions, which did not present a topographically useful spatial relationship to the HF, were not considered in this study.

### Protocol for segmentation

The intra- and extra-hippocampal locations of landmarks followed our previously published protocols for the adult and infant HF (Franko et al., 2014; Insausti & Amaral, 2012; Insausti et al., 1998a). B riefly, and proceeding in a rostrocaudal direction, the LI was taken as a starting point. The beginning of the EC is located at the level of the LI (landmark 1), about 1 mm in front of the start of the A (landmark 2). The LV (landmark 3) is located underneath the A body, at a slightly variable distance. The segment LI–LV roughly corresponds to the anterior half of the EC. Following in a caudal direction, the subiculum occupies the rostral limit of the LV—recognizable as an oblong structure inside the LV cavity, which forms the most anterior part of the hippocampal head (usually at mid-level of the A). The hippocampal head extends from the beginning of the subiculum as far as the caudal end of the uncus at the beginning of the LGN (landmark 4) marked by the gyrus intralimbicus, which indicates. This marks the end of the hippocampal head and thus the beginning of the hippocampal body. The hippocampus often shows hippocampal digitations during the perinatal period. However, we did not consider these a landmark since, in the best of cases, they are not visible until the midportion of the hippocampal head. The beginning of the LGN is roughly coincident with the caudal part of the EC—making it valuable as an extrahippocampal landmark. The separation between the body and tail of the hippocampus cannot be identified neither on MRI or histology, since their macroscopic and microscopic morphologies are similar. Therefore, we used the end of the LGN as a proxy for their differentiation. However, as it is not always possible to visualize the caudal limit of the LGN, an equally close boundary between the body and tail of the hippocampus is established at the level where the posterior pole of the Pul becomes visible (landmark 5). The end of the tail was established at the point where the obliquely cut fimbria turns dorsally to join the ventral aspect of the splenium of the corpus callosum (landmark 6) (Insausti et al., 2010). FieldsThe CA1 and CA3 fields of the hippocampal head and the dentate gyrus are not visible on our MRI images.

### Histological analysis

Nissl-stained series were analyzed in an optical Nikon Eclipse 80*i* microscope. Microphotographs were taken at low magnification in a densitometer (MCID, Paris, France) with a coupled camera. Brightness and contrast were adjusted using the Adobe® PhotoShop® CS 8.0.1 and Canvas X Build 925 (Deneba) software.

Distances among landmarks were calculated based on the section thickness of the images and the interval between adjacent sections. The LI was taken as a starting point in all cases, since the temporal pole was not always present.

### Statistical analysis

Statistical analysis was carried out using SPSS Statistical Software Package 19.0 (SPSS, Inc., Chicago, Illinois, U.S.). The normal distribution was verified using the Shapiro–Wilk test. Furthermore, Levene’s test was used to confirm the equality of variances. A paired t-test (p < 0.05) was employed to assess the MRI and histological data. Although the data showed a normal distribution, the low number of cases examined required performing a non-parametric Mann–Whitney test to support the results of the parametric test. Moreover, to verify the accuracy of each landmark’s measurement, we examined the differences between the values obtained between the rostrocaudal landmarks (LI–A, A–LV, LV–LGN, LGN–Pul, and Pul–Fx) in both techniques. A univariate ANOVA was performed for this purpose, with a p-value of 0.05 as the threshold for statistical significance.

## Results

Nine perinatal brain samples were analyzed both in MRI and Nissl-stained histological sections (Table 1). Distances between the six landmarks, obtained from both MRI and Nissl-stained histological sections, are presented in Table 2.

Length values in the youngest cases (case 1 with 33.5 gestational weeks (gw) and 6 days of life; and case 2 with 36 gw and 3 h of life) were the lowest, both in Nissl and MRI series. While the MRI total hippocampal length (LV–Fx) ranged between 13.2–25.2 mm, histological values ranged between 11.75–23.5 mm, thus showing a reasonably good agreement regarding the full length of the HF (12.0 vs. 11.75 mm on MRI and histology, respectively) (Table 2). However, the distances of the head (LV–LGN), body (LGN–Pul), and tail (Pul–Fx) of the hippocampus revealed some differences between MRI and histology. In the histological sections, the youngest cases (cases 1 and 2) showed the lowest hippocampal head and body distance values in the group (taking the LI as starting point). However, tail values were similar across cases. The lowest length values for the hippocampal tail were found in cases 4 (40 gw) and 5 (40 gw).

MRI landmark identification showed some variability. The anterior half of the EC (LI–LV) presented the greatest variability as assessed in both methods (Table 2). The hippocampal head showed the lowest values (cases 1 and 2). Body length values were relatively heterogeneous, with the lowest value corresponding to the youngest case (case 1). The tail of the hippocampus showed the most homogeneous values, although case 1 also had the lowest value of the group.

**Fig. 3:**
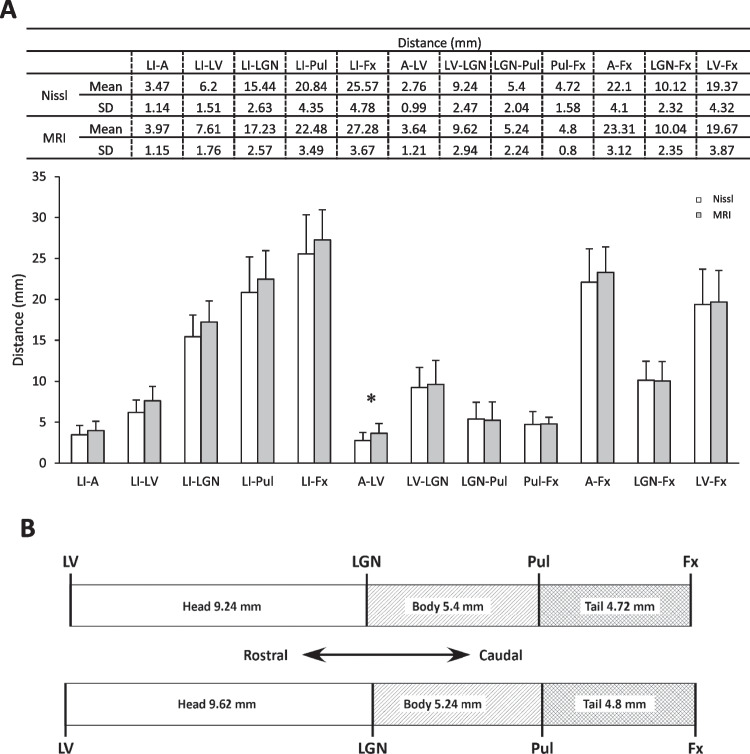
**A**: Mean values (± SD) and representation of data between landmarks obtained from MRI and histological analysis. White bars: histological sections; gray bars: MRI. *p < 0.05. **B**: Graphical representation of the distances corresponding to the segments of the hippocampus—head (LV–LGN), body (LGN–Pul), and tail (Pul–Fx)—obtained from histological sections (upper part) and MRI (lower part)

Figure 3A shows the mean values obtained by both MRI and histology. Statistical differences were observed only for A–LV (MRI: 3.64 ± 1.21; Nissl: 2.76 ± 0.99; p = 0.035). In addition, MRI values were slightly higher than in the histological series in most of the distances. The total length of the hippocampus itself (LV–Fx) was very similar on the MRI and histological series. A slightly larger difference was observed in the total HF length, although this was just 1.71 mm (27.28 ± 3.67 mm in MRI series versus 25.57 ± 4.78 mm for histology). The values of the head, body, and tail of the hippocampus were similar in both series (Fig. 3B).

**Fig. 4:**
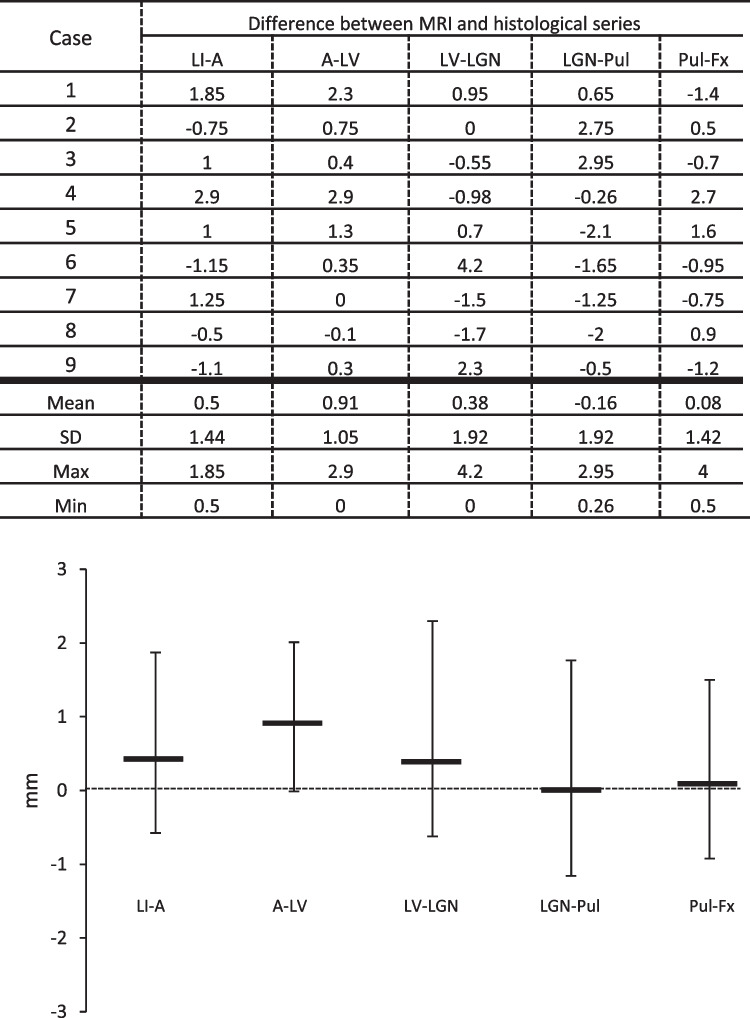
Upper part: differences between values obtained from MRI and histological analysis. Lower part: graphical representation of mean values from the data shown in the upper part. No statistically significant differences were obtained between the two methods of study

Distances between consecutive rostrocaudal landmarks were compared in MRI and histology (Fig. 4). The most significant difference between both series was observed in the hippocampal head (4.2 mm in case 6) and body (2.75 and 2.95 mm in cases 2 and 3, respectively). Cases with the shortest gestational age (cases 1 and 2) resulted in similar results compared to the other cases. The statistical analysis did not show significant differences. The differences between values obtained from MRI and histological analysis are available in the Supplementary Figs. 1.

## Discussion

The ex vivo MRI of fixed brain tissue offers an opportunity to identify anatomical landmarks pertinent to the HF in the medial temporal lobe. Those landmarks can be verified through histological analysis, and thus are potentially useful for in vivo, prenatal MRI (Brisse et al., 1997; Deshpande et al., 2015; Judas et al., 2005; Kostovic et al. 2002; Rados et al. 2006). However, the lack of maturity of the HF at the time of birth, as well as the limited resolution of the scans analyzed, prevented any annotation of hippocampal subfields—as has been done in other studies in adults (DeKraker et al., 2021; Iglesias et al., 2015; Yushkevich et al., 2021). A similar approach to landmark reliability has been performed in postnatal (Insausti et al., 2010) and adult cases (Augustinack et al., 2014; Franko et al., 2014; Insausti et al., 1998b). The examination of those landmarks in clinical situations (i.e., hypoxic–ischemic patients) could be of use in the assessment and prognosis of the pathology.

Our work provides a comparative, validated analysis of both ex vivo MRI and fine-grain histological study of the HF landmarks and length in normal newborns. Moreover, we show that these landmarks, similar to those used in the adult human HF (Franko et al., 2014; Insausti, ), might provide an accurate orientation for HF annotations at this early age.

Similar values were obtained with both methods of analysis—histology and MRI—with the exception of the A–LV distance. This discrepancy might be due to individual differences in the size and/or shape of the ventricular cavity in the temporal horn of the LV, that may render difficulthindering the identification of the anterior tip of the LV—which is extremely easy in histological series. Besides, another factor is the low resolution of the A and uncertainty in the localization of its precise starting point in newborn MRI scans. Otherwise, both MRI and histological length measurement methods yielded generally similar results, thus validating the use of in vivo MRI landmarks in clinical examinations.

The total length of the HF, represented by the LI–Fx distance, is about 5 cm in adulthood (Insausti & Amaral, 2012). Our results show that the length of the HF at 40 gw is nearly 3 cm, according to the perinatal group data (Fig. 3). This suggests that the postnatal growth of the HF almost doubles (2 cm) to reach the adolescent HF size by adolescence (around 14 years of age). Hippocampal growth shows considerable regional variability (Giedd et al. 1996b; Gilmore et al., 2007, 2012). Our results show that the mid-level of the LGN is approximately 1.7 cm at birth (40 gw), and therefore the postnatal growth of the LI–LGN distance (EC and head of the hippocampus) reaches about 1.5 cm. The length of the body and tail of the hippocampus (LGN–Fx) is close to 1 cm at birth, so it increases by about 1 cm by adulthood (Insausti & Amaral, 2012).

Different reports have analyzed brain growth patterns—either in whole brains or specific structures—mainly in children below the age of 4 years (Giedd et al., 1999; Giedd et al. 1996a; Hu et al., 2013). Knickmeyer et al. (2008) point to the rapid growth in the first year as a critical period in which the disruption of the developmental process may have long-lasting effects on brain structure and function. Most of the growth in brain size takes place after birth. In fact, brain volume increases from approximately 25% of the adult volume at birth to 95% by age 2; the hippocampus volume, in contrast, increases only by 13% (Knickmeyer et al., 2008; Pfefferbaum et al., 1994). The specific growth of the hippocampus is more significant during the first year of life—its volume increases by up to 80% during this period (Gilmore et al., 2012).

Most of the studies on the postnatal development of the human HF are performed in vivo, either during the neonatal period or during the first months of life (Gilmore et al., 2007, 2012; Knickmeyer et al., 2008). Other studies focus specifically on volumetric changes of the hippocampus in children starting at 8 (Schumann et al., 2007) or 13 years of age (Suzuki et al., 2005). However, little is known about the growth of the HF along the longitudinal axis. Our study also validates the use of extrahippocampal landmarks on MRI as more invariant to intrahippocampal lesions. Our estimations of the lengths of different segments of the HF in control children could contribute to establishing a standard pattern to compare with pathological situations.

However, this study has some limitations both from a technical and an anatomical point of view. For a more complete study, it would be necessary to have T1- and T2-weighted sequences. However, when this is not possible due to limitations in the equipment available at different medical centers, and despite the low contrast between gray and white matter, the protocol developed and presented in this work allows identifying the external profile of MRI images. Our protocol allows the identification of HF bumps and prominences in T1-weighted sequences at 1.5 T as specific portions or fields (i.e., the rostral subiculum), despite the low resolution of the MRI images. Furthermore, the analysis of histological serial sections validated the identification of specific gyri and portions of the hippocampus and parahippocampal gyrus. The comparison of measurements between low-field MRI and histological samples suggests high consistency in terms of the assessment of major landmarks.

Anatomical limitations are due to the curved profile of the HF. The hippocampus is indeed a dorsoventrally and medially oriented structure (Insausti et al., 2010). Thus, it is impossible to show any portion of the hippocampus in a single section plane—coronal, parasagittal, or horizontal. That is one reason why it was deemed necessary to add some anatomical significance to MRI images, even at low resolution as in the present case—since those images were obtained years ago, with the clinical MRI technology available at the time in the country. Nowadays, centers with 3 T scanners will be able to identify the external landmarks described as references in our work more precisely, allowing better study of the HF.

On the other hand, considering the LV as a landmark could lead to an overestimation of the hippocampal size. The LV appears as a small slit close to the amygdala, followed a few millimeters behind by the beginning of the subiculum. This small distance has been observed not only in developing but also in adult brains (Insausti & Amaral, 2012; Insausti et al., 2010). In the present work, the profile of the LV has been used as a proxy for the start of the hippocampus with the tangential section of the hippocampal head—which only contains the subiculum in the adult brain (Insausti & Amaral, 2012). The subiculum is not always visible as a rounded, oblong structure because of a lack of maturity. Given the subiculum’s lack of myelination (and contrast) during the perinatal period, the LV/start of the subiculum could serve as a proxy for the start of the hippocampus. A further consideration is that this is the only landmark enclosed in the temporal lobe, while all others have a profile that could help in the identification of subsequent landmarks.

The present work, taking advantage of the anatomical substrate of MRI images and the correspondence between landmarks and specific points in the HF, aims to facilitate interpretation by presenting a protocol fit for that purpose. It is important to validate protocols that are commonly used in adult or pediatric cohorts. Our proposal, focused on HF structures, considers that, if they are visible on low-resolution images, the use of higher magnetic fields (3 T or 7 T) would allow more precise estimations of length values of the HF longitudinal axis and the detection of possible differences between control and pathological cases.

## Conclusions

The identification of intra- and extrahippocampal landmarks on MRI series for comparison with histological series is a reliable method to analyze the complete longitudinal axis of the HF and segments of it in perinatal infants. This guide could be of use in clinical MRI studies of human postnatal development, where the standard length development of different HF portions could be compared with pathological situations to assess morphological changes—in volume or other aspects—associated with those disorders.

## Supplementary Information

Below is the link to the electronic supplementary material.

Supplementary file1 (PPTX 44 KB)** Supplementary Fig. 1:** Differences between values obtained from MRI and histological analysis

## Data Availability

All data generated or analyzing during this study are included in this published article (and its supplementary information files).

## Abbreviations

A: Amygdaloid complex
EC: Entorhinal cortex
Fx: Fornix
gw: Gestational weeks
HF: Hippocampal formation
LGN: Lateral geniculate nucleus
LI: *limen insulae*. Frontotemporal junction
LV: Lateral ventricle
Pul: Pulvinar complex of thalamus
