# Supplementary material for: Study of the human hippocampal formation: a method for histological and magnetic resonance correlation in perinatal cases
Source: Brain Imaging Behav. 2023 Apr 6;17(4):403–13. doi: 10.1007/s11682-023-00768-4 (PMC10435394; doi:10.1007/s11682-023-00768-4)
Supplement: Supplementary file 1 — Supplementary file1 (PPTX 44 KB) Supplementary Fig. 1: Differences between values obtained from MRI and histological analysis [file 11682_2023_768_MOESM1_ESM.pptx]

## Slide 1
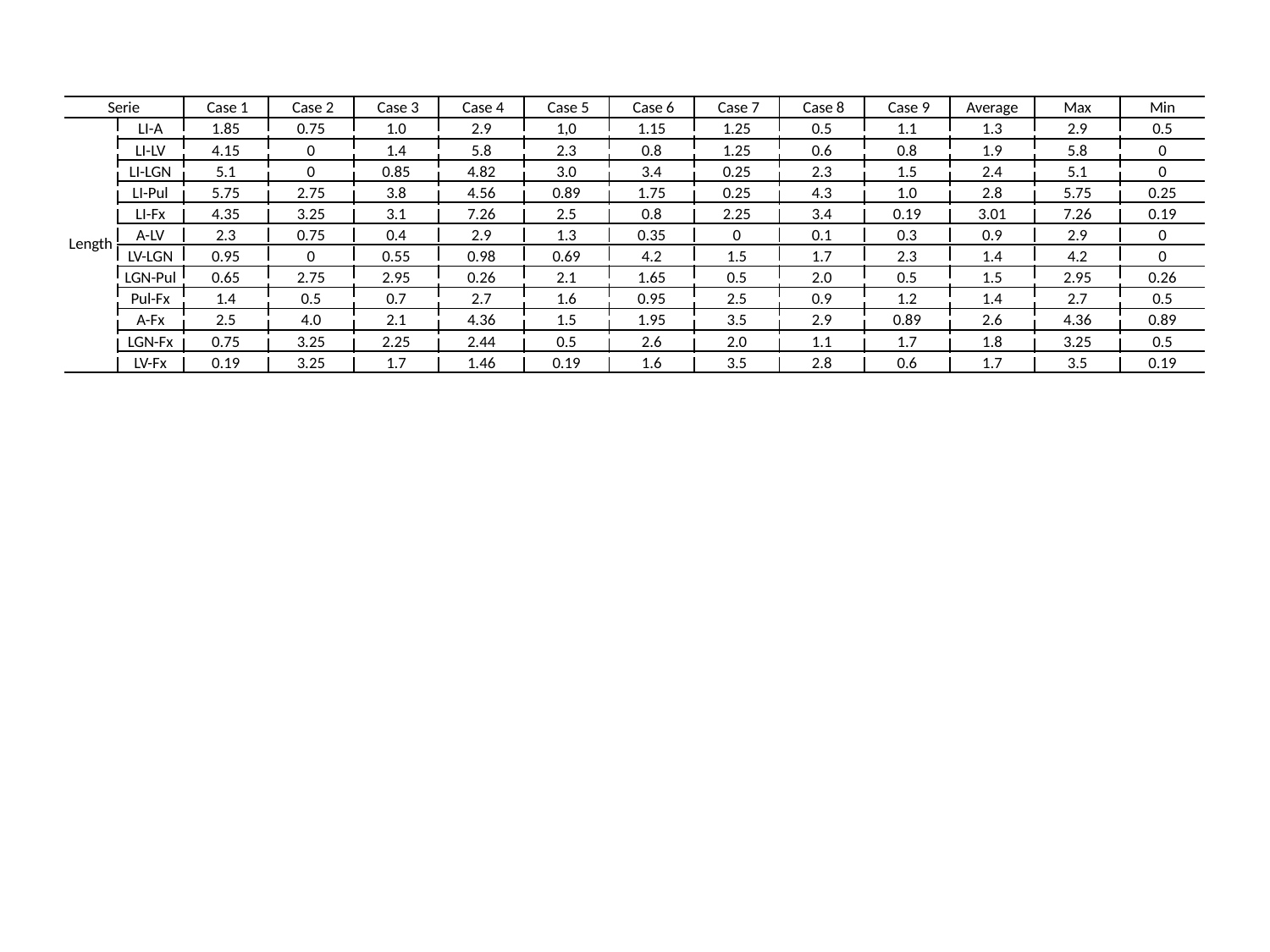

| Serie | | Case 1 | Case 2 | Case 3 | Case 4 | Case 5 | Case 6 | Case 7 | Case 8 | Case 9 | Average | Max | Min |
| --- | --- | --- | --- | --- | --- | --- | --- | --- | --- | --- | --- | --- | --- |
| Length | LI-A | 1.85 | 0.75 | 1.0 | 2.9 | 1,0 | 1.15 | 1.25 | 0.5 | 1.1 | 1.3 | 2.9 | 0.5 |
| | LI-LV | 4.15 | 0 | 1.4 | 5.8 | 2.3 | 0.8 | 1.25 | 0.6 | 0.8 | 1.9 | 5.8 | 0 |
| | LI-LGN | 5.1 | 0 | 0.85 | 4.82 | 3.0 | 3.4 | 0.25 | 2.3 | 1.5 | 2.4 | 5.1 | 0 |
| | LI-Pul | 5.75 | 2.75 | 3.8 | 4.56 | 0.89 | 1.75 | 0.25 | 4.3 | 1.0 | 2.8 | 5.75 | 0.25 |
| | LI-Fx | 4.35 | 3.25 | 3.1 | 7.26 | 2.5 | 0.8 | 2.25 | 3.4 | 0.19 | 3.01 | 7.26 | 0.19 |
| | A-LV | 2.3 | 0.75 | 0.4 | 2.9 | 1.3 | 0.35 | 0 | 0.1 | 0.3 | 0.9 | 2.9 | 0 |
| | LV-LGN | 0.95 | 0 | 0.55 | 0.98 | 0.69 | 4.2 | 1.5 | 1.7 | 2.3 | 1.4 | 4.2 | 0 |
| | LGN-Pul | 0.65 | 2.75 | 2.95 | 0.26 | 2.1 | 1.65 | 0.5 | 2.0 | 0.5 | 1.5 | 2.95 | 0.26 |
| | Pul-Fx | 1.4 | 0.5 | 0.7 | 2.7 | 1.6 | 0.95 | 2.5 | 0.9 | 1.2 | 1.4 | 2.7 | 0.5 |
| | A-Fx | 2.5 | 4.0 | 2.1 | 4.36 | 1.5 | 1.95 | 3.5 | 2.9 | 0.89 | 2.6 | 4.36 | 0.89 |
| | LGN-Fx | 0.75 | 3.25 | 2.25 | 2.44 | 0.5 | 2.6 | 2.0 | 1.1 | 1.7 | 1.8 | 3.25 | 0.5 |
| | LV-Fx | 0.19 | 3.25 | 1.7 | 1.46 | 0.19 | 1.6 | 3.5 | 2.8 | 0.6 | 1.7 | 3.5 | 0.19 |
